# Body Mass Index (BMI) and Waist Circumference Monitoring in Psychiatric Inpatient Setting and Staff Knowledge on Monitoring and Management of These Parameters

**DOI:** 10.1192/bjo.2026.11481

**Published:** 2026-06-30

**Authors:** Rajasee Sharma, James Sutton, Praveen Kumar

**Affiliations:** 1 Birmingham and Solihull Mental Health Foundation Trust, Birmingham, United Kingdom; 2 Blackcountry Healthcare NHS Foundation Trust, Dudley, United Kingdom

## Abstract

**Aims:**

BMI and waist circumference should be measured on admission and then regularly for inpatients. We aimed to determine whether this was being met, and to explore staff knowledge of such monitoring.

**Methods:**

Retrospective data was collected from Rio, the electronic records system at Bushey Fields Hospital. The fifteen most recently discharged patients from each of five wards as of April 2025 were extracted, after which six patients from each set of fifteen were selected at random. Monitoring data was then extracted by two reviewers.

A questionnaire was separately distributed amongst ward staff to assess staff knowledge of BMI and waist circumference monitoring.

**Results:**

30 patients were included, with ages ranging from 20 to 81, comprising 18 male and 12 female patients. 26 patients were taking an antipsychotic. Patients had a range of physical comorbidities and were taking a range of additional medications.

BMI was recorded at admission for 22 patients, at one week for 20 patients, at two weeks for 23 patients, and at three weeks for 20 patients. Waist circumference was recorded at admission for 9 patients, and at one week for 29 patients.

13 patients had an above normal BMI, but action was only taken for 4. This included lifestyle advice, provision of relevant leaflets and a referral to appropriate services.

15 staff members received the questionnaire, of which 12 responded. 100% of responders were aware of the normal BMI range, but only half knew the normal range for waist to height ratio. 92% were aware of the need for weekly monitoring for patients on antipsychotics, but only 33% were confident in recording relevant parameters on Rio.

Only 25% of responders felt confident referring patients to relevant services, and 75% felt that educational resources on the ward were inadequate.

**Conclusion:**

Not all inpatients on psychiatry ward receive necessary BMI and waist circumference monitoring, and appropriate management is only initiated for a small number of patients.

Most survey responders were not confident recording relevant information on RiO or making referrals to appropriate services and felt that available educational resources were inadequate.

We have made several suggestions in light of this data, including updating electronic record, introducing posters to the ward, staff refresher workshops and the introduction of a streamlined referral pathway for dietician reviews.

